# Influence of online comments on clothing impulse buying behavior in mobile short video app live broadcast

**DOI:** 10.3389/fpsyg.2022.913073

**Published:** 2022-08-11

**Authors:** Tian Hewei

**Affiliations:** Fashion Department, Xiamen Academy of Arts and Design, Fuzhou University, Xiamen, China

**Keywords:** elaboration likelihood model, online comments, impulse buying behavior, fashion involvement, live broadcast

## Abstract

Based on elaboration likelihood model (ELM), this paper introduces the central route and peripheral route of online comments and constructs a conceptual model affecting consumers’ clothing impulse buying behavior in live broadcast. A total of 737 questionnaires were collected, and 709 valid questionnaires were used for questionnaire analysis. According to the ELM, there are central route (comment quality and comment comprehensiveness) and peripheral route (comment quantity and commentator credibility) of online comments. The results show that in addition to the commentator credibility, the comment quality, comment comprehensiveness, and comment quantity have a significant positive impact on impulse buying behavior. Fashion involvement plays a moderating role in the relationship between online comments and impulse buying behavior. This paper will provide theoretical support for live broadcast clothing marketing and provide suggestions for the development and design of live broadcast.

## Introduction

According to the 47th statistical report on China’s Internet Development released by China Internet Network Information Center (CINIC), China’s online retail sales reached 11.76 trillion yuan in 2020, an increase of 10.9% over 2019. Among them, the online retail sales of physical goods amounted to 9.76 trillion yuan, accounting for 24.9% of the total retail sales of social consumer goods. By December 2020, the number of online shopping users in China had reached 782 million, an increase of 72.15 million over March 2020, accounting for 79.1% of the total number of Internet users. It can be seen that online shopping has become one of the most common forms of consumption. During online shopping, consumers cannot contact real goods and can only make purchase decisions through the product information displayed online (Von et al., 2018). Online comments are third-party information that consumers can refer to in the decision-making process in addition to the information displayed by merchants. Therefore, online comments have become another important source of information for consumers to buy online (Cho and Chan, 2021).

In recent years, a new form of online shopping, mobile short video app has been promoted in China, and live broadcast of mobile short video app is loved by more and more people (Kim et al., 2021). With the development of mobile short video app, mobile shopping malls are gradually opened for marketing through live broadcast (Mhalla et al., 2020). Douyin is the originator of online shopping of mobile short video app in China, and then, many online shopping malls have begun to launch live broadcast marketing. In 2021, the sales of several major mobile short video app in the double 11 shopping festival reached more than 360 billion US dollars. At present, there are many studies on social media marketing. Haenlein et al. (2020) had conducted a study of influence marketing and analyzed how to enhance the impact through Instagram, TikTok, and Co. Nair et al. (2022) believed that TikTok improves user satisfaction and forms user participation, brand effect, and effective promotion and suggested that entrepreneurs should first use social media applications that can provide user satisfaction for marketing. Comp et al. (2020) believed that TikTok will be the next social media frontier. Although China’s Douyin mobile short video app (MSVA) business has been successful, there are few studies on it, and there is no study on impulse buying behavior of clothing in mobile short video app live broadcast.

Therefore, this paper attempts to use ELM theory, take the comment quality (CQ) and comment comprehensiveness (CC) as the central route and the comment quantity (CQ1) and commentator credibility (CC1) as the peripheral route, and construct a conceptual model affecting the clothing impulse buying behavior in mobile short video app live broadcast. The model takes the central route and peripheral route as independent variables, fashion involvement as a regulatory variable, and impulse buying behavior as a dependent variable. This paper is roughly organized as follows. First, this paper combs the literature on ELM theory, online comments, impulse buying behavior, and fashion involvement and puts forward the conceptual model and research hypothesis. Second, it expounds the research methods used in this paper and discusses the research results. Finally, the conclusions, suggestions, and limitations of this paper are summarized.

The main contributions of this paper are as follows: First, based on the theory of information processing, this paper examines the mediating role of consumer attitudes and conformity psychology in the relationship between online comments and purchase intention. This paper applies the information processing theory to the domestic network marketing management research earlier, which further enriches the research perspective of the “black box” exploration of network marketing theory. Second, this paper tests the effectiveness of online comments in the context of China and improves the understanding of online marketing management from the perspective of system innovation. Finally, this paper tests the influence of online comments on the formation of consumer attitudes and conformity psychology, further enriches the research results of antecedents of consumer attitudes and conformity psychology, and provides the reference for the practice of enterprise online marketing management.

## Theoretical background

### Elaboration likelihood model theory

Elaboration likelihood model (ELM) is a theoretical model proposed by Petty and Cacioppo that has the most profound impact in the field of consumer information processing (Petty and Cacioppo, 1986). The model believes that the process of individual adoption of information is a persuasive process. When consumers receive information, they will make a series of judgments on the reliability of information, which can further affect their decision making on information adoption (Ho and Bodoff, 2014). Different types of information processing processes are represented as the central route and peripheral route in ELM theory (Cacioppo et al., 1986). In the central route, consumers are interested, motivated, and able to process information. They will seriously consider the arguments of information and evaluate the attributes of information arguments, so as to change their attitude, change their purchase intention, and make purchase decisions. There is also another kind of consumers who lack interest, motivation, and ability in information processing, and their attitude, purchase intention, and purchase decision will be affected by the peripheral route (Liao and Huang, 2021).

The ELM systematically reveals the process of consumer information adoption (Chang et al., 2020). The online comments seen when buying clothing in MSVA are essentially a kind of information. The adoption process of these online comments is similar to that of consumer information. Both online comments and information need to be reviewed and processed before the adoption relationship can be finally established. Shahab et al. (2021) believed that the ELM can explain the impact of promotional information and content marketing on consumer attitudes and behavior. Wang and Lee (2019) used the ELM to distinguish the central route and peripheral route in the process of Chinese consumers accepting new SNS products. The results show that both central route factors and peripheral route factors have a significant impact on the purchase intention. Dep (2021) used the ELM to study the purchase intention of millennials in Sri Lanka and found that online customer comments had a positive impact on consumers’ purchase intention. Tyas and Hutagaol (2021) believed that ELM can explain the impact of online comments on social media on Muslim fashion e-commerce website Hijup, and the research results showed that online comments posted by Facebook and Instagram all have a positive impact on clothing sales in Hijup. These studies have verified the impact of ELM in social media marketing. Using ELM theory, this paper attempts to explore the impact of central route and peripheral route on the clothing impulse buying behavior of Chinese mobile short video app live broadcast.

### Online comments

Online comments (Chen et al., 2022), online reviews (Zhang et al., 2021), and electronic word of mouth (Sanjeev and Neha, 2021) refer to the content that consumers express their subjective feelings about the sold goods or services through a specific online shopping platform by using online Internet technology. Most online reviews are comments on products or services. Such comments are after consumers purchase products or enjoy services, personal thoughts, opinions, or feelings written and published for products or services are usually text-based SMS. With the rapid development of e-commerce, the role and influence of online reviews are very important. Fang (2022) believed that consumers’ online purchase behavior is a consumption decision under the condition of incomplete information, and this random decision determines the sales volume of product online sales. Therefore, online comments are very important for consumers. The online comments in this paper refer to the personal views of consumers on the release of clothing products, when using mobile short video app live broadcast, and also refer to the evaluation released for the purchased clothing after purchasing the clothing in mobile short video app live broadcast.

Steven (2016) believed that online reviews significantly affect ‘consumers’ consumption intention and consumption decision making, and highly praised online reviews can promote online transactions. Lee et al. (2021) believed that online reviews provide an important information reference for consumers’ online shopping. Consumers’ online reviews share personal opinions and experience, which can promote a multi-directional interaction between sellers and consumers and consumers and consumers, and online reviews are an important source of information feedback mechanism of online trading platform. Wu et al. (2020) believed that high-quality online review information has an important impact on users’ purchase behavior and can promote the transformation of consumers’ shopping intention and the change in shopping behavior. Seo and Lee (2013) investigated the impact of the direction and content of online comments on consumers’ attitudes toward clothing products, and the results showed that positive and objective online comments lead to a higher level of consumer attitude. Kim and Kim (2018) studied consumers’ responses to online reviews of sports shoes, and the study found that when consumers have shopping plans, online reviews can improve their purchase intention, while when consumers have no shopping plans, online reviews have no significant impact on their purchase intention. These studies are aimed at the traditional online shopping mall, the online comments in mobile short video app live broadcast have changed, and it can produce dynamic, instantaneous, and responsive online comments. This paper attempts to explore the impact of these new online comments on clothing impulse buying behavior.

### Impulse buying behavior

Rook (1987) believed that impulse buying refers to an instantaneous, overwhelming, and continuous purchase desire experienced by consumers without purchase plan and purchase consciousness. Beatty and Ferrell (1998) believed that impulsive purchase refers to immediate purchase without any shopping goal, whether it is to buy a specific product category or meet a specific demand. Impulsive purchase occurs after consumers experience their purchase desire without much reflection. Han et al. (1991) described the fashion-oriented impulse as a suggestion impulse, that is, the purchase is based on the self-suggestion of buying new fashion products. In fashion-oriented impulse buying, consumers have not experienced new fashion products before. Impulse buying behavior in this paper refers to the purchase behavior of clothing without any plan when consumers use mobile short video applications to watch live broadcast, stimulated by online comments, hosts, and other factors. Impulse buying is a very important factor in clothing marketing.

Cengiz (2017) studied the influence of fashion pursuit on purchase decision and impulse purchase and analyzed 333 samples with Amazon shopping experience. The results showed that the need for popularity had a positive impact on clothing purchase decision and impulse buying behavior and purchase decision had a positive impact on impulse buying behavior. Wiranata and Hananto (2020) believed that online shopping leads to irrational behaviors such as impulse buying and analyzed the influencing factors of clothing impulse buying and it explores the impact of website quality on impulse buying. The results showed that website quality has no positive impact on clothing impulse buying and sales promotion and fashion consciousness had a positive impact on impulse buying. Chen et al. (2021) investigated the impact of Internet celebrities on luxury fashion brand impulse purchase. Through the survey of 585 Internet celebrity fans in China, they found that trust is an important factor affecting impulse buying. The sense of identity and perceived fit also significantly promote impulse buying through trust, but a large social distance may damage the relationship between trust and impulse buying. These studies have paid attention to the important role of impulse buying in fashion marketing, but there is a lack of research on mobile short video application shopping. Therefore, this paper carries out the research on clothing impulse buying of mobile short video application live broadcast.

### Fashion involvement

Traylor (1981) believed that product involvement refers to the importance of products to consumers. It refers to not only the correlation between personal values and products, but also the willingness and dependence of individuals on products. Zaichkowsky (1994) believed that involvement refers to the importance or relevance of purchase decisions perceived by individuals on the basis of their needs, interests, and values, and the antecedents of participation are personal characteristics, object characteristics, and situational characteristics, which are considered to affect consumers’ participation in advertising, products, and purchase decisions. In order to evaluate the personal involvement and consumer involvement scale, Goldsmith and Emmert (1991) used the multi-clue and multi-method matrix to verify the convergence and discriminant effectiveness of the scale. Product involvement includes consumers’ awareness, demand, and favor of products. The higher the consumers’ awareness of products, the higher the involvement of products. In this paper, fashion involvement refers to the consumers’ understanding of fashion products, as well as their enthusiasm, interest, and participation in purchasing fashion products.

Gitimu et al. (2013) believed that consumers’ evaluation of clothing quality is affected by fashion involvement. Through multivariate analysis of variance and analysis of variance, it is confirmed that fashion involvement significantly affects consumers’ evaluation of clothing quality. Hidayat and Tryanti (2018) studied the impact of fashion involvement and shopping lifestyle on impulse buying behavior. Through multiple linear analysis, the partial test results showed that fashion involvement has a negative significant impact, while the simultaneous test results showed that both variables have a positive significant impact on impulse buying behavior. Padmasari and Widyastuti (2022) discussed the impact of fashion involvement, shopping lifestyle, and promotion on impulse purchase. The statistical analysis used was the multiple linear regression. The results showed that in Shopee e-commerce, fashion involvement, shopping lifestyle, and promotion significantly affected the impulse buying of fashion products. Hewei and Youngsook (2021) studied the satisfaction and loyalty of China’s Post-00 generation in fashion shopping applications. The results showed that fashion involvement played a regulatory role in the impact of fashion shopping application characteristics on satisfaction and loyalty. These studies have confirmed the important role of fashion involvement in clothing marketing. Whether it is offline shopping or online shopping, fashion involvement has an impact on purchase behavior. Therefore, this paper takes fashion involvement as an adjustment variable, to explore the adjustment role of fashion involvement in the impact of online comments on clothing impulse buying behavior.

## Research model and hypotheses

**FIGURE 1:**
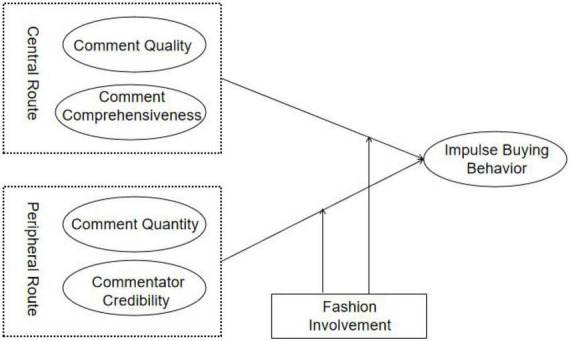
Concept model.

As a persuasion model, ELM theory was widely used in the study of attitude, social communication, and consumer behavior. In the past literature, this model has been used to explore the research of consumers’ online consumption behavior and attitude. At present, live broadcast, as a new type of marketing rising all over the world, is different from traditional online consumption. We try to explore the mode of consumers’ consumption attitude and behavior in live broadcast based on ELM theory. This study constructs ELM of the impact of online comments on impulse buying behavior, as shown in Figure 1. The concept model is composed of central route and peripheral route. The central route is the comment quality and comment comprehensiveness of online comments and the core clue of online comments. The peripheral route is the comment quantity and the commentator credibility, and it is the marginal clue of online comments. In addition, there is a close relationship between fashion involvement and consumers’ information involvement in fashion products. Generally speaking, the higher the fashion involvement, the higher the familiarity with fashion product information. Fashion involvement may affect the relationship between online comments and impulse buying behavior. Based on this, this paper considers the moderating effect of fashion involvement in the relationship between online comments and impulse buying behavior.

### Central route

According to ELM theory, when consumers have high motivation and ability to process information, the central route plays a role, and consumers will spend more time and energy to evaluate and judge information and form a stable attitude. High-quality comments will directly have a positive impact on consumer behavior. The comprehensiveness of online comments means that there are both positive and negative comments made by consumers. The comprehensiveness of comments will make consumers have higher information processing motivation. Aghakhani et al. (2021) believed that the central path and peripheral path of the ELM determine the usefulness of online comments. When the central path is consistent with consumers’ own opinions, it can have a positive impact on the usefulness of adoption of online comments. Deng et al. (2021) examined the role of consumers’ initial trust in the persuasion process of Chinese e-commerce advertising, and they believed that under the tendency of low trust, the quality of online comments had a significant positive impact on purchase intention. Zhang et al. (2018) believed that the usefulness consumers feel from online comments will enhance browsing behavior. Browsing will positively affect purchase impulse and ultimately affect consumers’ impulse buying behavior. Based on this, this paper puts forward the following hypotheses:

**H1:** The comment quality in the live broadcast of mobile short video applications has a positive impact on impulse buying behavior.

**H2:** The comment comprehensiveness in the live broadcast of mobile short video applications has a positive impact on impulse buying behavior.

### Peripheral route

According to ELM theory, when consumers’ willingness and ability to process information are low, the peripheral route plays a role. Consumers will simply process information according to the number of text comments, the number of picture or video comments, and the reviewer’s credit. They are more vulnerable to the influence of opinion leaders and show a herd mentality. The more the online comments, the higher the popularity and attention of the product, which affects the consumer’s herd consumption psychology. The reviewer’s credibility refers to the reviewer’s professional ability and reliability, as well as the information receiver’s trust in the reviewer or the acceptance of his comments. A higher number of comments and the credibility of commentators will reduce consumers’ processing motivation. Zhang et al. (2022) believed that by participating in real-time online comments, consumers feel a strong sense of social existence, and the rolling real-time online comments have a great impact on consumers’ perceived value and purchase decisions. Andoy et al. (2022) believed that online comments have become an important source for consumers to obtain product information, and the credibility of online comments affects consumers’ purchase intention that can create a virtual atmosphere, which significantly affects online impulse buying behavior. Based on this, this paper puts forward the following hypotheses:

**H3:** The comment quantity in the live broadcast of mobile short video applications has a positive impact on impulse buying behavior.

**H4:** The commentator credibility in the live broadcast of mobile short video applications has a positive impact on impulse buying behavior.

### Fashion involvement

Fashion involvement is the subjective feeling of consumers about the relationship between fashion products and themselves, which is affected by consumers’ own needs, value judgments, and preferences. Strong, pleasant, and personalized purchase experience has a positive impact on consumers’ involvement in fashion products. Online comment can promote the communication and contact between consumers and consumer groups of fashion products and services and then strengthen consumers’ conformity psychology and value recognition. A stable and continuous interaction during live broadcast can strengthen consumer needs and preferences and promote the formation of consumption habits. A good live consumption experience can improve transaction efficiency, reduce transaction costs, and enable consumers to gain more value perception, thereby enhancing fashion involvement. Lee and Rhee (2018) examined the two-way interaction between online word-of-mouth effect (acceptance and redelivery intention) and fashion involvement and market maven. The results showed that fashion involvement affected online word-of-mouth acceptance, while market maven mainly affected redelivery intention. Haq et al. (2014) studied the relationship between impulse buying, hedonism, and fashion involvement. The results showed that fashion involvement plays a regulatory role in the relationship between hedonism and impulse buying. Andani and Wahyono (2018) identified the influence of promotion, hedonic shopping motivation, fashion involvement, and positive emotion as intervention variables on impulse purchase. The results showed that promotion, hedonic shopping motivation, and fashion involvement affected impulse purchase. Chae et al. (2016) believed that the impression formed by online comments has a positive impact on the intention of word-of-mouth communication, thus affecting the brand attitude, and fashion involvement played a regulatory role in word-of-mouth communication. Based on this, this paper puts forward the following hypotheses:

**H5:** Fashion involvement plays a moderating role in the relationship between central route and impulse buying behavior.

**H6:** Fashion involvement plays a moderating role in the relationship between peripheral route and impulse buying behavior.

## Materials and methods

### Scale development

Our research was approved by the academic committee and ethics committee of Fuzhou University (202109072). In order to ensure the validity of the questionnaire, based on the literature review, this study excavated the measurement indicators that meet the variables in the study. All measurement items were determined from previous e-commerce or mobile commerce research and adapted to the background of mobile short video applications live broadcast, as given in Table 1.

**TABLE 1:** Research measurement scale.

Dimension	Variable name	Measurement index system
Central route	Comment quality (CQ)	Q1: The comments truly reflect the purchase experience. Q2: The comments truthfully reflect the product attributes. Q3: The comments are objective and rational on the whole.
	Comment comprehensiveness (CC)	Q4: The comments include the advantages and disadvantages of the product. Q5: The comments cover many aspects of products and services. Q6: Comprehensive description through text, pictures, and videos.
Peripheral route	Comment quantity (CQ1)	Q7: There are a lot of comments on this fashion product. Q8: There are many positive comments on this fashion product.
	Commentators credibility (CC1)	Q9: I think the commentators know the product very well. Q10: I think the commentators on this product are very trustworthy.
Impulse buying behavior	Impulse buying behavior (IBB)	Q11: The comments attract me to continue watching. Q12: The comments precipitate me to buy clothes. Q13: The comments make me think irrationally.
Fashion involvement	Fashion involvement (FI)	Q14: I think fashion products are very important to me. Q15: I am very concerned about fashion information.

The central route includes the comment quality and comment comprehensiveness. With reference to the research scale of Zhang et al. (2018) and Aghakhani et al. (2021), a total of six questions were retained. The peripheral route includes comment quantity and commentators credibility. Referring to the research scale of Floh and Madlberger (2013) and Zhang et al. (2018), a total of four questions were retained. The latent variable of impulse buying behavior refers to the research scale of Han et al. (1991) and Wiranata and Hananto (2020), and three questions were retained. Fashion involvement refers to the research scale of Haq et al. (2014) and Chae et al. (2016), and two questions were retained.

The questionnaire was measured by a five-point Likert scale, in which 1 means completely disagree and 5 means fully agree. Before the formal issuance of the questionnaire, we conducted a pilot test to determine whether the survey instruments were understandable for participants and any ambiguous or confusing measurement items.

### Data collection and sample

**FIGURE 2:**
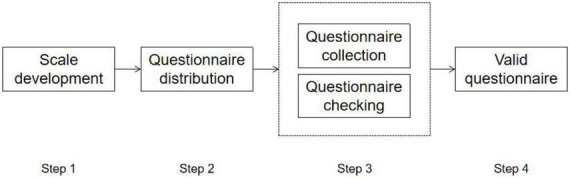
Questionnaire collection process.

We collected data through a professional online survey website.^1^ To motivate the respondents, we gave a digital currency of 1–5 RMB randomly. During 2 weeks of data collection, we collected 737 samples in total. We carefully removed the questionnaires with more than five missing values and those with the same answer to all questions. Besides, we also checked the IP address to avoid multiple answering from one respondent. After data cleaning, we yielded 709 samples with a valid rate of 96%. The questionnaire collection process is shown in Figure 2.

The data collection time was from June 21, 2021, to July 4, 2021. The research object of this study is the people who have the experience of purchasing fashion products online. The effectiveness of the research object is ensured by 2 methods, namely, the design of questionnaire description and the distribution channel of questionnaire. Send the QR code pictures of the online survey questionnaire to WeChat groups, online communities, and forums where online shopping fashion products are concentrated, and clearly mark the QR code pictures with identifying words such as “consumption experience of fashion products” and “mobile short video app shopping of clothing.”

As given in Table 2, there are 418 women with consumption experience in fashion products, accounting for 58.56%, 488 people aged from 26 to 35 years, accounting for 68.76%, and 527 people with monthly disposable income of more than 5,000 RMB, accounting for 74.33%. It can be seen that the consumption objects of fashion products are mainly young people with a high average disposable income every month. The survey results show that all respondents have experience in buying clothes in mobile short video applications. Their most popular mobile shopping app is Douyin.

**TABLE 2:** Characteristics of social population structure.

Characteristic variable	Demographic	Frequency	Percentage (%)
Gender	Male	291	41.44
	Female	418	58.56
Age	< 18	45	6.35
	18–25	126	17.82
	26–30	269	37.94
	31–35	219	30.82
	>35	50	7.07
Monthly disposable income (RMB)	<5,000	182	25.71
	5,001–10,000	314	44.27
	10,001–15,000	186	26.18
	15,001–20,000	27	3.84
	> 20,000	12	2.79

1$≈6.68 RMB.

## Results and discussion

### Reliability and validity

Reliability test mainly measures the internal quality of the model. First, this study uses combined reliability (CR), mean variance extraction (AVE), and Cronbach’s α to test the reliability of six main latent variables. The test results are given in Table 3. The CR of the six main latent variables is above 0.8. The mean variance extraction values were greater than 0.5. The coefficients of Cronbach’s α are greater than 0.8, indicating that the survey data have good reliability.

**TABLE 3:** Reliability test of the scale.

Latent variable	Items	CR	AVE	Cronbach’s α
Comment quality	3	0.883	0.649	0.886
Comment comprehensiveness	3	0.874	0.715	0.903
Comment quantity	2	0.826	0.673	0.853
Commentator credibility	2	0.895	0.694	0.917
Impulse buying behavior	3	0.853	0.761	0.889
Fashion involvement	2	0.872	0.6569	0.916

For the test of aggregation validity, the AVE can be used. It refers to how much of the variation of potential variables comes from the measurement index. The larger the AVE, the higher the interpretation degree of the measurement index to the latent variable, which proves that the measurement index designed by the latent variable has good convergence. Generally, when the AVE of each potential variable is close to 0.5 or more than 0.5, it indicates that the measurement model has good aggregation validity. The test of discriminant validity can be judged by the square root of AVE. If the square root of AVE is greater than the correlation coefficient between the latent variable and other latent variables, it indicates that the common variance between the latent variable and its own measurement index is greater than that with other latent variables. Different latent variables have obvious differential validity in the measurement index. When the square root of AVE of each latent variable meets this condition, it can show that the measurement model has good discriminant validity.

As given in Table 4, in terms of aggregate validity and discriminant validity test, the AVE of each factor is greater than 0.5, and the arithmetic square root of AVE value is greater than its correlation coefficient with other factors, indicating that the scale of this study has good aggregate validity and discriminant validity.

**TABLE 4:** Validity test of the scale.

	CQ	CC	CQ1	CC1	IBB	FI
CQ	0.742					
CC	0.529	0.775				
CQ1	0.671	0.596	0.736			
CC1	0.566	0.473	0.529	0.782		
IBB	0.624	0.538	0.461	0.458	0.816	
FI	0.525	0.475	0.537	0.683	0.586	0.724

### Structural equation model analysis

The fitting index results of structural equation model are given in Table 5. It can be seen from Table 5 that *x*^2^/df is 3.24, between 2 and 5, meeting the judgment criteria. The RMR value is 0.026, which meets the judgment standard of “less than 0.05.” The RMSEA value is 0.038, which meets the judgment standard of “less than 0.08.” The values of GFI, NFI, and CFI are 0.931, 0.926, and 0.914, respectively, which meet the judgment standard of “greater than 0.9.” Each fitting index meets the requirements, indicating that the fitting degree of the model is good.

**TABLE 5:** Goodness-of-fit test of the scale.

*x* ^2^	*x*^2^/df	RMR	RMSEA	CFI	GFI	NFI
226.52	3.24	0.026	0.038	0.931	0.926	0.914

AMOS is used to conduct structural equation model analysis on the collected data to obtain the path coefficient between various variables to verify the hypothetical relationship. The results are given in Table 6. Due to the good fitting of the assumed model, it can be judged from the path coefficient in Table 6 that the comment quality, comment comprehensiveness, and comment quantity of clothing in mobile short video app live broadcast have a positive and significant impact on the impulse buying behavior (the standardized path coefficient being 0.316, 0.104, and 0.252, respectively, and *P* < 0.001), and hypotheses H1, H2, and H3 are supported. The influence of commentator credibility on impulse buying behavior is not significant (the standardized path coefficient being 0.114, *P* = 0.264), and hypothesis H4 is not supported.

**TABLE 6:** Model’s path inspection index.

Hypothesis (Paths)	Estimate	S.E.	*t*-value	*P*	Results
CQ→IBB	0.316	0.152	3.286	***	Supported
CC→IBB	0.104	0.128	2.538	***	Supported
CQ1→IBB	0.252	0.087	4.314	***	Supported
CC1→IBB	0.114	0.159	2.671	0.264	Not supported

*p < 0.05, **p < 0.01, ***P < 0.001.

### Moderating effect test

This study uses the regression equation to test the moderating effect, and Model 1 makes a regression analysis on the impact of core and marginal approaches on purchase intention. Model 2 makes a regression analysis on the influence of central route, peripheral route, and fashion involvement on purchase intention, and Model 3 makes a regression analysis on the influence of independent variables, moderating variables, and mutual moderating items on purchase intention.

The analysis results are given in Table 7. The value of *R*^2^ changes from 0.026 of Model 1 to 0.042 of Model 2 (increased by 0.016) and then to 0.063 of Model 3 (increased by 0.021). The Δ*F*-value changes from 0.003 of Model 1 (*P* = 0.000) to 0.032 of Model 2 (*P* = 0.000) and then to 0.027 of Model 3 (*P* = 0.000). The *R*^2^ value and *F*-value are less than 0.05, which indicates that there is a moderating effect, and hypotheses H5 and H6 are supported.

**TABLE 7:** Test results of moderating effect of fashion involvement.

Variables		1st model		2nd model		3rd model	
		β	*t-*value	β	*t-*value	β	*t-*value
Independent variables	CQ	0.261	3.582	0.142	2.471	0.107	1.314
	CC	0.149	2.384	0.073	1.166	0.054	0.762
	CQ1	0.527	5.396	0.238	3.182	0.183	2.086
	CC1	0.108	2.145	0.026	1.025	0.018	0.779
Moderating variables	IBB			0.431	1.883	0.315	1.253
Interaction effect	CQ*IBB					–0.117	–2.184
	CC*IBB					–0.032	–1.073
	CQ1*IBB					–1.613	–4.195
	CC1*IBB					–0.028	–0.072
*R*^2^(Δ*R*^2^)		0.026 (0.026)		0.042 (0.016)		0.063 (0.021)	
Δ*F*-Value(p)		0.003 (*p* = 0.000)		0.032 (*p* = 0.000)		0.027 (*p* = 0.000)	

*Indicates the relationship between the two variables.

## Discussion

Based on ELM theory, this study divides the impact of online comments of clothing on impulse buying behavior in mobile short video app live broadcast into central route (comment quality, comment comprehensiveness) and peripheral route (comment quantity, commentator credibility), discusses the impact relationship between the central route and peripheral route on impulse buying behavior, and introduces the concept of fashion involvement, to explore the moderating effect of fashion involvement in the relationship between the central route and peripheral route of online comments on impulse buying behavior. The specific research discussion is as follows:

The central route of online comments, comment quality, and comment comprehensiveness has a significant positive impact on impulse buying behavior. The influence path coefficient of comment quality on impulse buying behavior was 0.316 (*P* < 0.001), and the influence path coefficient of comment comprehensiveness on impulse buying behavior was 0.104 (*P* < 0.001). This shows that when consumers buy clothing through mobile short video app live broadcast, objective and rational comments that truly reflect their purchase experience and product attributes can have a positive impact on consumers’ impulse buying behavior. At the same time, they fully introduce the advantages and disadvantages of products and are able to evaluate products and services from multiple perspectives such as text, pictures, or videos, which can positively affect consumers’ impulse buying behavior. This finding is consistent with Hong’s research results. Hong et al. (2021) believed that in Malaysia, online comments quality directly affects consumers’ online impulse buying behavior. Zhao et al. (2020) believed that consumers’ online shopping behavior is affected by other consumers’ online comments and comment comprehensiveness affected purchase intention, and it is also consistent with our research results.

The comment quantity of peripheral route has a significant positive impact on impulse buying behavior, but the influence of commentator credibility on impulse buying behavior is not significant. The influence path coefficient of the comment quantity on impulse buying behavior was 0.252 (*P* < 0.001), and the influence path coefficient of commentator credibility on impulse buying behavior was 0.114 (*P* = 0.264). This shows that when consumers buy clothing through mobile short video app live broadcast, the comment quantity on clothing, especially the number of positive comments, can more positively affect consumers’ impulse buying behavior. Zafar et al. (2021) believed that a large number of online comment interactions affect impulse buying behavior, but the authenticity of comments plays a negligible role in impulse buying. This is consistent with our results, and we believe when the peripheral route plays a role, it is relatively difficult for consumers to judge the commentator’s credibility. At the same time, fashion products are generally low-cost consumables, and consumers pay more attention to their perceived entertainment value when purchasing. Therefore, the influence of commentator credibility on impulse buying behavior is not significant.

Fashion involvement has a moderating effect on the central route and peripheral route of online reviews. According to the connotation theory of involvement, the higher the fashion involvement of consumers, the more they can analyze and judge the online comments, and consumers are more aware of the value that the products will bring to themselves, so as to improve the impact of online comments on purchase intention, while consumers with low fashion involvement reduce the impact of online comments on purchase intention. Choirul and Artanti (2019) believed that positive emotions can no longer affect the impulse buying behavior of millennials and fashion involvement can affect impulse buying behavior and regulate the impact of store atmosphere and hedonic shopping motivation on impulse buying behavior. This is consistent with our research results. In mobile short video live broadcast, fashion involvement can affect impulse buying behavior and regulate the relationship between other variables as an intermediary variable.

Furthermore, from the perspective of psychology, consumers’ impulse buying behavior depends on consumers’ fine processing of received information. When consumers can consciously think about MSVA messages carefully, after careful thinking, analysis, and induction of the target information, it will eventually lead to impulse buying behavior. When consumers do not actively think about MSVA messages, the positive or negative aspects of MSVA messages, or technical hints, may lead to impulse buying behavior. The higher the degree of participation, the more detailed the overall understanding, so as to influence impulse buying behavior in a central way. When consumers’ motivation and ability to analyze information are low, their psychology and emotions are more vulnerable to the influence of external things, thus affecting impulse buying in peripheral ways. On the contrary, mobile short video can create a relaxed and pleasant environment. Short video content is usually funny, exaggerated, and desirable, so in this case, consumers are not willing to spend energy thinking about short video information, thus affecting impulse buying behavior through peripheral paths.

## Conclusion and implications

This study explores the path and mechanism of the impact of online comments in mobile short video app live broadcast on impulse buying behavior and obtains the empirical data support. The results show that when consumers are exposed to online comments of fashion products, they will judge the online comment information through the central route and peripheral route and finally affect their impulse buying behavior. The comment quality, comment comprehensiveness, and comment quantity of online comments can positively affect the impulse buying behavior. Fashion product sellers in mobile short video app live broadcast should make efforts in these aspects to guide buyers to make some objective, rational, and specific comments on the attributes or service characteristics of fashion products, as well as comprehensive comments combined with text, pictures, and videos. At the same time, businesses can also take some measures to improve the enthusiasm of consumers’ comments, so as to realize the positive impact of online comments on consumers’ impulse buying behavior.

Based on this research, MSVA can design more effective systems and short video works to make consumers more impulsive. Practitioners need to pay more attention to consumers’ motivation and ability of information processing in the shopping process of mobile short video applications. For example, the design of the system should improve the two-way communication. At present, the two-way communication function of MSVA is not perfect, and the effectiveness of information communication between consumers and the MSVA platform cannot be guaranteed. At the same time, the platform should ensure the high quality of short video content, which is the key factor to harvest traffic and comments. In addition, the platform should encourage consumers to participate in interactive exchanges, encourage consumers to publish high-quality and surprising comments, and attract more consumers’ attention. Short video producers should pay attention to the comprehensibility and emotional resonance of the video content and create short videos that can arouse consumers’ positive emotions to resonate with consumers.

## Limitations and future scope

Based on ELM theory, this paper has certain theoretical value on the impact of online comments on impulse buying behavior, but there are still limitations. For example, only four aspects of online comments are selected as independent variables, which is relatively lack of more comprehensive consideration. The research sample selects people who have the experience in purchasing fashion products in mobile short video app live broadcast. The research object is only for online comments in mobile short video app live broadcast. These problems affect the comprehensiveness and depth of the research to a certain extent. This work does not take expert comments as a variable to study, but is also limited to consumers. If these elements are included, the model of this paper will have stronger explanatory power and higher reference value. In our research, some assumptions are not supported. We conclude that the existing theories cannot reasonably explain consumers’ e-commerce behavior in the emerging technology environment, and many concepts and phenomena are still hidden. In future research, we need to introduce more different variables and carry out more in-depth research on different consumer groups, and we also suggest that other researchers further draw a new theory of modern consumer e-commerce consumption behavior from qualitative research.

## Data availability statement

The raw data supporting the conclusions of this article will be made available by the authors, without undue reservation.

## Ethics statement

The studies involving human participants were reviewed and approved by the Academic Committee and Ethics Committee of Fuzhou University (202109072). Written informed consent from the patients/participants or patients/participants legal guardian/next of kin was not required to participate in this study in accordance with the national legislation and the institutional requirements.

## Author contributions

TH completed data collection, analysis and manuscript writing.

## Footnotes

1
www.wjx.cn
